# Testing the test strips: laboratory performance of fentanyl test strips

**DOI:** 10.1186/s12954-023-00921-8

**Published:** 2024-01-18

**Authors:** John C. Halifax, Lilly Lim, Daniel Ciccarone, Kara L. Lynch

**Affiliations:** 1 Department of Laboratory Medicine, ZSFG Clinical Laboratory, UCSF, 1001 Potrero Ave. Bldg. 5 2M16, San Francisco, CA 94110 USA; 2 Department of Family and Community Medicine, University of California San Francisco, 500 Parnassus Avenue, MU-3E, Box 900, San Francisco, CA 94143 USA

**Keywords:** Fentanyl test strips, Harm reduction, Drug checking, Overdose, Substance use, Opioids

## Abstract

**Background:**

The overdose crisis driven by synthetic opioids continues to escalate in the USA. We evaluated the efficacy of multiple manufacturing lots of a fentanyl test strip (FTS) to detect fentanyl and fentanyl analogs and assessed cross-reactivity with possible interferences.

**Methods:**

Drug standards were dissolved in water in a laboratory setting and serially diluted. Drug dilutions were tested using five different manufacturing lots of BTNX Rapid Response (20 ng/mL cutoff) lateral flow chromatographic immunoassay strips to assess lot-to-lot variability for FTS sensitivity and cross-reactivity for the analytes of interest.

**Results:**

All five manufacturing lots cross-reacted with fentanyl and eleven fentanyl analogs. Diphenhydramine, lidocaine, MDMA, and methamphetamine were found to cause false positives with the strips. There was notable lot-to-lot variability in the sensitivity of the strips for fentanyl, fentanyl analogs, and known interferences.

**Discussion:**

FTS remains an important overdose prevention tool, but lot-to-lot variability in performance complicates robust instructions that balance the prevention of false positives and false negatives. Continued lot-to-lot performance assessment is recommended to ensure health education for FTS remains accurate. More sophisticated drug checking technologies and services are needed in the community landscape to augment personal FTS use to facilitate informed consumption and overdose risk mitigation.

**Supplementary Information:**

The online version contains supplementary material available at 10.1186/s12954-023-00921-8.

## Background

The triple wave epidemic of overdose deaths due to prescription opioids, heroin, and illicitly manufactured fentanyls (IMF) in the USA has reached historic proportions [1]. In the USA in 2021, 80,411 deaths were attributed to opioids with a rate of 24.7/100,000 US population [2]. The fastest growing overdose wave is due to synthetic opioids other than methadone—largely IMF, i.e., fentanyl and fentanyl analogs—accounting for 70,601 reported deaths in 2021, representing a 1,173% increase from 2014[2].

Drug seizure data highlight rising levels and varieties of IMF. In 2019, the number of fentanyl reports submitted to the National Forensic Laboratory Information System (NFLIS) were more than double those for heroin [3]. Beginning in 2014, deaths attributed to IMF have risen alongside seizures [4–6]. US Northeast and Midwest regions were initially more heavily affected by fentanyl-related overdose [6, 7]; however, fentanyl is now present in the illicit substance supply in the western states on par with national availability [3], with a subsequent rise in fentanyl-related deaths west of the Mississippi [8]. The chemical family of fentanyls is growing, and while the majority of fentanyl-related overdose is attributed to the main chemical, fentanyl analogs are significantly contributing to overdose deaths. In the highly impacted region of 10 US states, fentanyl analogs were detected in overdose toxicology in 5,083 (19.5%) of 26,104 examined overdose deaths [9]. Eleven different fentanyl analogs and synthetic opioids have been identified in recent drug seizures [10], although hundreds are known to exist and more theoretically possible [11, 12]. In relation to morphine, fentanyl is 100 times as potent by weight and thus estimated to be 40 times more potent than heroin[13]. There is a 4-log range of potencies for the fentanyl analogs: from 1.5 to 10,000 times that of morphine [14, 15].

Previously, most IMF available in the USA came in the form of fentanyl-adulterated or fentanyl-substituted heroin [16]. Persons who use fentanyl-adulterated or fentanyl-substituted heroin are often unaware of the adulteration and have mixed opinions about its desirability [16–18]. Those with experience can discern fentanyl-adulterated or fentanyl-substituted heroin from heroin with several strategies, but the utility of this is unknown [16]. Recent trends, however, indicate that heroin is being replaced by fentanyl as the dominant opioid in the illicit substance supply [19, 20]. In addition to heroin, IMF has been found in counterfeit opioid and benzodiazepine pills [6, 21–23]. Increasing exposure to IMF among stimulant users (e.g., cocaine and methamphetamine) has been noted in both screening [24] and post-mortem toxicology studies [25].

Greater surveillance for IMF in the illicit substance supply is recommended to address the US crisis [13]. Point-of-use drug checking has been used in Europe and Australia to inform users of potential contamination of their substances [26–29]. A range of testing options suitable for harm reduction services are available [28]. Rapid testing for fentanyl exists as a urine immunoassay, which can be adapted to direct drug testing. These fentanyl test strips (FTSs) have emerged as a harm reduction strategy albeit with a number of challenges [16, 30–33]. Early findings on use of FTS among US community-based samples reveal acceptability [34, 35] and significant positive changes in reported drug use behavior following a positive fentanyl test [36–38]. As perceptions of fentanyl ubiquity become increasingly common in much of the USA, there is a risk the incentive for such positive behavioral changes decreases as fentanyl exposure is considered unavoidable by people who use drugs [32, 36]. However, even when fentanyl is considered unavoidable, precluding positive impacts at the individual level, people who use drugs still describe FTS as a useful tool at the community level [39].

There has been rapid implementation of FTS in several US locations, e.g., NY, MD, DE, CA [38, 40]. Current use in drug checking contexts differs in terms of sample preparation; dissolution of drugs in water as prepared for injection, re-hydration of drug residue post-preparation, and dissolution of a portion of a drug sample to be consumed undissolved are all performed and likely produce varying drug concentrations in solution. The most widely available FTS, distributed by BTNX [41], has sensitivity parameters developed for urine drug screening not direct drug testing. BTNX claims sensitivity for qualitative detection of fentanyl and its metabolite norfentanyl in urine at a cutoff concentration of 20 ng/mL [42]. They further claim ability to detect multiple fentanyl analogs including carfentanil, acetyl fentanyl, butyryl fentanyl, remifentanil, ocfentanil, sufentanil, p-fluoro fentanyl, furanyl fentanyl, valeryl fentanyl, and 3-methyl fentanyl [42].

There have now been several independent scientific assessments of BTNX (20 ng/mL cutoff) FTS for use in drug checking. As summarized in Table 1, these studies demonstrate a variance in the effective cutoff concentration for true positive detection of fentanyl, with only one assessment approaching the manufacturer reported limit of detection of 20 ng/mL [41–46]. The presence of methamphetamine and MDMA in high concentrations has been shown, by only one study, to cause false positives with the BTNX FTS [45], leading some community organizations to advocate for diluting samples to 2 mg/mL to avoid false positives [47]. The corresponding concern in diluting samples is that with unreliable limits of detection for fentanyl and its analogs, the pursuit of minimizing false positives may lead to false negatives, particularly among stimulant users who may be opioid naïve. Such concern is heightened as the overdose crisis enters a “fourth wave” characterized by increasing deaths involving stimulants, which may be a result of stimulant contamination with fentanyl, co-use, or both [48]. However, assuming the reported 20 ng/mL detection limit is accurate, diluting a homogenized drug sample to 2 mg/mL would allow the FTS to detect fentanyl present in the sample down to 0.001% purity. This level of sensitivity is likely more than adequate, even in opioid naïve users, assuming that other risk reduction strategies are also utilized.

**Table 1:** Summary of results from previous BTNX rapid response fentanyl test strip (20 ng/mL cutoff) evaluations

Study	Year	Fentanyl limit of detection (ng/mL)	Fentanyl analog cross-reactivity	Interferences
Green et al.	2020	100	2 of 2 assessed cross-reacted	Not Assessed
Bergh et al.	2021	50	25 of 28 assessed cross-reacted	None found among substances assessed
Lockwood et al.	2021	25	Not Assessed	Methamphetamine, MDMA, Diphenhydramine
Wharton et al.	2021	100	19 of 29 assessed cross-reacted	Not Assessed
Park et al.	2022	200	13 out of 17 assessed cross-reacted	Not Assessed

Variation in the performance of BTNX FTS makes the standardization of FTS instructions difficult and may undermine utility and user trust in FTS as a protective intervention. Recent community communication highlighted the poor performance of an individual lot of BTNX FTS and raised the concern of lot-to-lot manufacturing variability [49]. The current study is the first independent study to assess BTNX FTS across five different manufacturing lots, including lot 196, the lot reported by community advocates as defective. Limits of detection for fentanyl, a range of fentanyl analogs, and other chemical interferences were determined for each lot.

## Methods

### Standards, reagents, test strips

All analytical standards were purchased from Cayman Chemicals (Ann Arbor, MI) or Cerilliant Corporation (Round Rock, TX). Water used was analytical grade and purchased from Fisher Scientific. Drug-free human urine was purchased from Golden West Diagnostics (Temecula, CA). BTNX Rapid Response™ fentanyl test strips, 20 ng cutoff (sold for clinical use—part number FLY-1S48-100, referred to as BTNX-20), were obtained from BTNX (Markham, ON). Five different manufacturing lots of these test strips (D607082, 16,120,004, DOA2101018, DOA2111188, and DOA2105196, referred to as 082, 004, 018, 188, and 196 lots, respectively) were tested to assess lot-to-lot variability in performance. Lots 082 and 004 were obtained and evaluated in 2017, with lots 018, 188, and 196 obtained in 2021 and evaluated in summer 2022. The principal investigator was present for both rounds of testing to ensure continuity of methods. Both rounds of testing were performed in the same laboratory space. These test strips are lateral flow chromatographic competitive immunoassay tests [50].

#### Strip sensitivity and cross-reactivity

Fentanyl, fentanyl analogs and one non-fentanyl synthetic opioid, U-47700 were spiked into water at various concentrations (10 µg/mL, 5 µg/mL, 1 µg/mL, 500 ng/mL, 200 ng/mL, 100 ng/mL, 50 ng/mL, 20 ng/mL, and 10 ng/mL) and tested with the five different lots of test strips (in duplicate) according to the manufacturers’ recommendations. Assuming a 2 mg/mL dissolution of the drug samples, these fentanyl concentrations represent fentanyl present in the sample at purities ranging from 0.5 to 0.0005%. These purities are likely representative of street samples with low levels of fentanyl introduced to samples by poor handling of multiple substances in the drug supply chain. Briefly, individual test strips were immersed up to the max mark for the BTNX strips into each test vial containing the standards at specific concentrations. The test strips were held in the liquid for 10 s and then placed flat on a clean surface. Test strip results were photographed and interpreted according to manufacturer instructions within 5 min of test initiation independently by two different people. A result was concluded and recorded by consensus after discussion between the two reviewers.

#### Interferences

Pure analytical standards of illicit substances, common adulterants, and cutting agents were diluted in water to various concentrations to evaluate non-specific binding and/or cross-reactivity with the BTNX 20 test strips. High upper concentrations (150 mg/mL if available) for these interferences were chosen to ensure some level of cross-reactivity was achieved. Additionally, since two of these analytes (methamphetamine and MDMA) are stimulants that may be used by opioid-naïve individuals who may want greater assurance of fentanyl absence, it was hypothesized that samples expected to be stimulants would be tested at higher concentrations. In some instances, it was not possible to evaluate concentrations greater than 10 mg/mL, 25 mg/mL, or 50 mg/mL due to the limited amount of pure analytical standard available for purchase from companies supplying drug standards to laboratories with DEA licenses. Test solutions containing standards were tested according to the same procedure as described above.

#### Urine and water eluent comparison

The use of FTS to assess fentanyl presence in drugs dissolved in water is an off-label use of immunoassay strips designed to detect fentanyl presence in human urine. The manufacturer reported 20 ng/mL cutoff referred to the cutoff in urine. FTS performance in drug-free human urine was assessed to establish a baseline of FTS performance when used on-label to compare to their performance when used off-label. Analytical fentanyl standard was spiked into drug-free human urine and water separately at various concentrations (1 µg/mL, 500 ng/mL, 200 ng/ml, 100 ng/mL, 50 ng/mL, 20 ng/mL) and tested with three different lots in duplicate. Test solutions containing standards were tested according to the same procedure as described above.

## Results

### Fentanyl test strip sensitivity and cross-reactivity

**Fig. 1:**
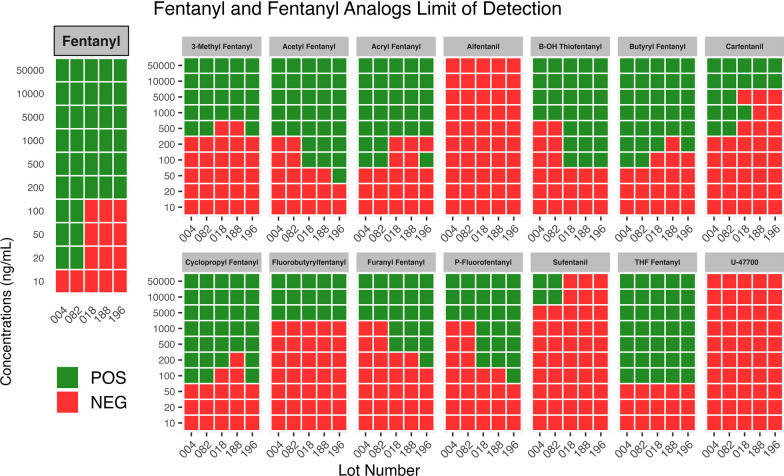
Fentanyl test strip reactivity with fentanyl and select fentanyl analogs. BTNX Rapid Response Fentanyl Test Strip (20 ng/mL cutoff) limits of detection for fentanyl and fentanyl analogs. POS indicates a positive result, NEG indicates a negative result

The five lots of BTNX Rapid Response™ fentanyl test strips (20 ng cutoff) evaluated showed cross-reactivity for the following fentanyl analogs: 3-methylfentanyl, acetyl fentanyl, acrylfentanyl, β-hydroxy-thiofentanyl, butyrylfentanyl, carfentanil, cyclopropylfentanyl, fluorobutyrylfentanyl, furanyl fentanyl, p-fluorofentanyl, and tetrahydrofuran fentanyl (see Fig. 1). The high degree of structural similarity between fentanyl and these fentanyl analogs can be seen in Additional file 1: Fig.S1). The two lots from 2017, 082 and 004, also detected sufentanil, while the three 2021 lots did not. None of the five lots detected alfentanil or U-47700 at any concentration, with these two synthetic opioids and sufentanil having more significant structural differences from fentanyl and the other analogs (see Additional file 1: Fig.S1).

Sensitivities for fentanyl and fentanyl analogs varied significantly across the five lots of BTNX test strips evaluated. The two lots evaluated in 2017, 082, and 004, detected fentanyl at the manufacturer reported cutoff of 20 ng/mL. The three lots obtained in 2021, 018, 188, and 196, were only able to detect fentanyl down to a lower limit of 200 ng/mL, an order of magnitude above the reported cutoff (see Fig. 1). Sensitivity for 3-methylfentanyl, fluorobutyrylfentanyl, and tetrahydrofuran fentanyl were approximately the same across all five lots of BTNX strips, with differences in sensitivity for other analogs largely falling along lines of whether the lot was sourced in 2017 or 2021. The three lots sourced in 2021 (018, 188, and 196) exhibited enhanced sensitivity for acetyl fentanyl, β-hydroxy-thiofentanyl, furanyl fentanyl, and p-fluorofentanyl, with lower limits of detection ten to fifty times lower than the 2017 lots. Alternatively, the three 2021 lots demonstrated poorer sensitivity relative to the 2017 lots for acrylfentanyl (with the exception of lot 196), butyryl fentanyl, and carfentanil (with lot 018 showing improved sensitivity relative to the other 2021 lots, but still inferior to the 2017 lots). Sensitivity varied between the three lots of 2021 sourced strips for multiple analogs, with particular variance for acrylfentanyl, carfentanil, and cyclopropylfentanyl (Fig. 1).

### Fentanyl test strip interferences

Cross-reactivity with other illicit drugs and potential cutting agents or adulterants was evaluated for the BTNX-20 test strips. For the two 2017 sourced lots, 082 and 004, heroin (10 µg/mL), 6-acetylcodeine (10 µg/mL), quinidine (10 µg/mL), cocaine (25 mg/mL), and ketamine (25 mg/mL) showed no degree of cross-reactivity or interference. Positive interferences were detected for diphenhydramine and lidocaine at 100 mg/mL. MDMA produced a positive result on the test strip at 50 mg/mL. Cross-reactivity with methamphetamine was stereospecific, with *l*-methamphetamine at 25 mg/mL producing a negative result and *d*-methamphetamine producing a positive result when tested at 5 mg/mL.

**Fig. 2:**
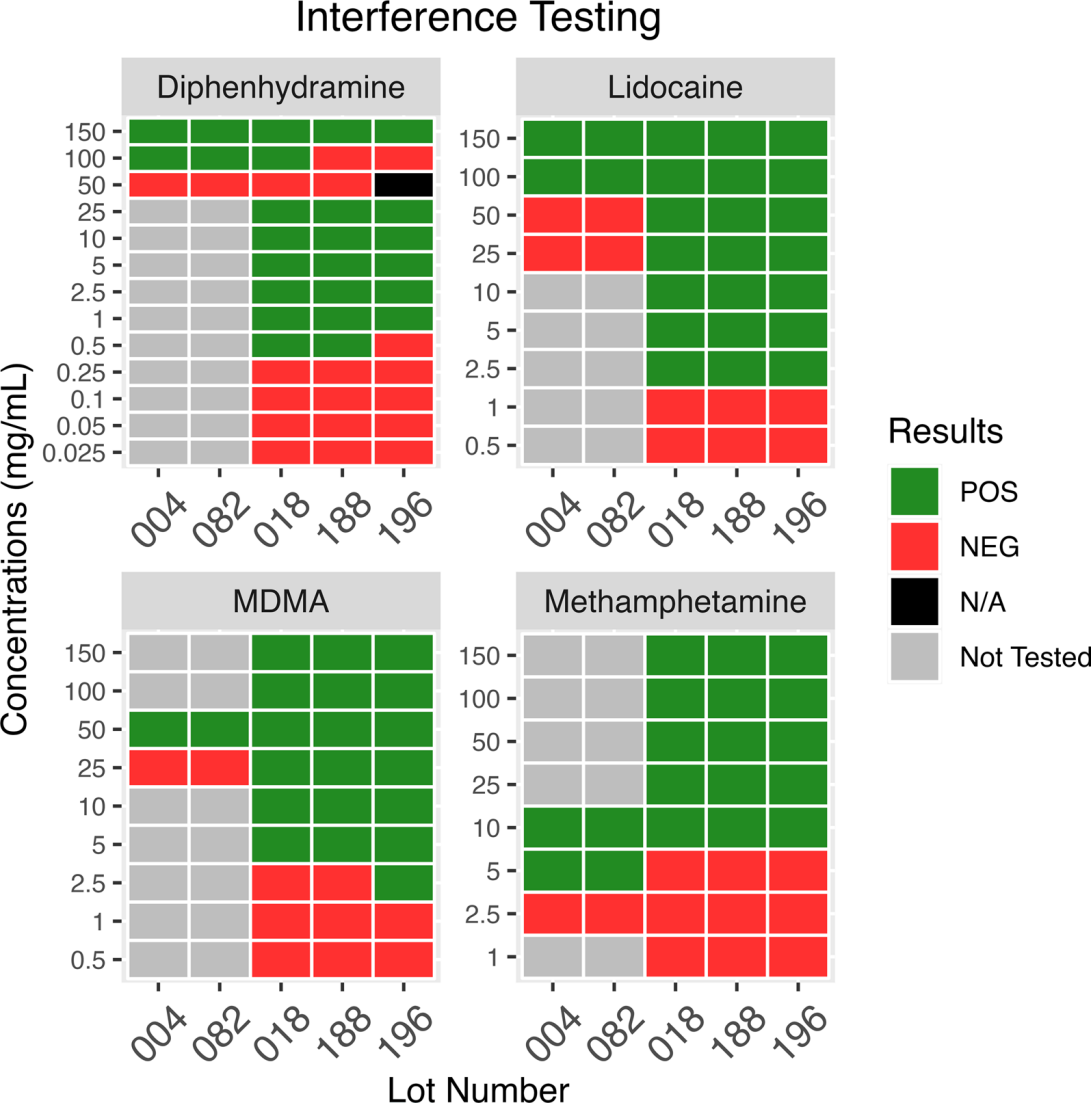
Fentanyl test strip reactivity with interferences. BTNX Rapid Response Fentanyl Test Strip (20 ng/mL cutoff) limits of cross-reactivity for interferences. POS indicates a positive result, NEG indicates a negative result, N/A indicates a lack of control line on multiple tests indicating an invalid test, and Not Tested indicates concentrations not evaluated

Follow-up interference testing was performed on the 2021 sourced lots on known interferences from the 2017 lots and literature. Lidocaine (2.5 mg/mL) and *d-*methamphetamine (10 mg/mL) produced false positives at the same cutoffs across all three lots. MDMA produced false positives at 5 mg/mL for lots 018 and 188, but at 2.5 mg/mL for lot 196. Levamisole, a common cocaine adulterant, showed no degree of cross-reactivity up to 100 mg/mL. Diphenhydramine, a common heroin adulterant, showed cross-reactivity at 0.5 mg/mL for lots 018 and 188 and 1 mg/mL for 196, but exhibited limited cross-reactivity at 50 and 100 mg/mL. This limited cross-reactivity at 50 and 100 mg/mL was characterized by unusually faint control lines and extremely faint to invisible test lines, resulting in consensus negative assessments at these concentrations (Fig. 2). These experiments were repeated, and the same unexplained results were observed. For lot 196, multiple different strips did not show a control line at 50 mg/mL, marked as “N/A” in Fig. 2.

### Urine and water as test strip eluent comparison

Test strip performance in drug-free human urine was assessed in the three 2021 sourced lots to explore whether the lower-than-expected sensitivity for fentanyl could be due to the use of water as test strip eluent. All three lots did perform better in urine than water, with two lots (018 and 196) having a lower cutoff of 50 ng/mL, and the third (118) having a lower cutoff of 100 ng/mL (Additional file 2: Fig. S2). Control and test lines were visibly brighter on urine-tested strips than strips tested on water solutions evaluated contemporarily, which reproduced fentanyl detection cutoffs found in previous testing.

## Discussion

The laboratory sensitivity testing of five BTNX-20 fentanyl test strips revealed findings that have important implications for real-world testing. From 2017 to 2021, the BTNX-20 strips decreased in sensitivity for fentanyl from 20 to 200 ng/mL, consistent with other laboratory testing trends in those time periods [41, 43–46]. This loss of sensitivity for fentanyl with the recent lots is concerning. None of the lots evaluated detected alfentanil or the non-fentanyl synthetic opioid U-47700, and the recent lots did not detect sufentanil. The U series of synthetic opioids is growing in member number and are regularly detected in the illicit substance supply, with U-47700 availability seeming to peak in 2017–2018 but remaining available [51]. The lower sensitivity of the 2021 lots for carfentanil is concerning, as this potent fentanyl analog has been noted in several deadly overdose outbreaks [52, 53]. Improved FTS performance for fentanyl in urine compared to water indicates that the manufacturer reported sensitivity cutoff of 20 ng/mL is likely urine-specific and cannot be extrapolated reliably to drugs dissolved in water, the generic scenario in harm reduction contexts. However, FTS cutoffs in urine solutions did not reproduce the manufacturer-reported cutoffs and were lot dependent, with the two best performing of three lots evaluated having a lower limit of 50 ng/mL.

Specificity testing found false positives for methamphetamine and MDMA, consistent with a previous evaluation [45]. This is an important finding as stimulant-only users may have greater interest in screening their drugs for fentanyl given their lack of tolerance for opioids. The finding that *d*-isomer of methamphetamine is falsely positive for fentanyl at lower concentrations is key, as the street supply of methamphetamine is predominately *d*-isomer of high purity [54]. Accurate fentanyl detection in stimulant samples is increasingly important as the overdose crisis enters a “fourth wave” characterized by an increase in stimulant-related overdoses [48]. The decrease in sensitivity for methamphetamine cross-reaction with BTNX-20 strips from 5 in the 2017 lots to 10 mg/mL across all 2021 lots is thus a gain in immunoassay selectivity, reducing the likelihood of methamphetamine induced false positives. However, the concentration threshold for MDMA false positives with the BTNX-20 strips decreased between the 2017 and 2021 lots, from 50 to 2.5–5 mg/mL. Whereas with 2017 lots, the cutoff for MDMA false positives was higher than likely sample concentrations for an FTS test (i.e., it is unlikely someone would dissolve a sample as high as 50 mg/mL for testing), the 2021 lots cause false positives between 2.5 and 5 mg/mL, a more realistic testing concentration. Similarly, lidocaine, a common adulterant of cocaine [55], and diphenhydramine, a common adulterant of opioids [56], gave false-positive BTNX-20 results at 100 mg/mL for the 2017 lots, with false positives occurring at more realistic testing concentrations of 2.5 and 0.5–1 mg/mL for the 2021 lots, respectively. However, at the higher concentrations of diphenhydramine (beyond realistic testing concentrations), we observed repeated paradoxical results with the 2021 lots.

These longitudinal changes in interference sensitivities highlight the need for continued assessment of new test strip lots, and the difficulty of providing a robust set of instructions for sample dilution prior to test strip use. Some harm reduction organizations recommend precise dilution guidelines for use of the BTNX-20 strips, for example, to dilute methamphetamine and MDMA down to 2 mg/mL to avoid false positives. These instructions, originally based on the results of interference testing with the 2017 lots, would now leave the strips vulnerable to a diphenhydramine or MDMA false positive with strips performing at the level of the 2021 lots. Similarly, given the lower fentanyl sensitivity for the 2021 lots compared to the 2017 lots, false negatives are possible with excessive dilution of fentanyl and fentanyl analogs below the limit of detection. Additional file 3: Table S1 A–D illustrates how longitudinal changes in lot-to-lot FTS performance while balancing false-positive and false-negative possibilities make determining an ideal sample concentration difficult.

While lot-to-lot variability should be considered, FTS remains sensitive to fentanyl and fentanyl analogs to create a broad window of concentrations between undesired interference cross-reactivity and desired fentanyl analog detection cutoffs. Drug checking samples in British Columbia confirmed by laboratory reference qNMR methods determined that FTS failed to detect fentanyl in 4 of 173 (2.3%) fentanyl-positive samples, with all four false-negative samples containing fentanyl at 5% concentration by weight or less [57]. These real-world results, produced using FTS with drug samples concentrations of approximately 30 μg/mL [58], indicate good FTS sensitivity performance at a low concentration that eliminates false positives. Expected opioids comprised 70% of samples tested, and only 9% were expected stimulants, limiting generalizability of FTS effectiveness when testing drugs other than opioids and when testing outside a fixed drug checking site with staff experienced with FTS use [57]. When instructing ideal FTS testing concentrations, public health education for FTS use should weigh the need to prevent false negatives for fentanyl and fentanyl analogs with the desire to avoid false positives with known interferences.

There are alternative portable technologies for screening for IMF including infrared spectroscopy and Raman spectroscopy [28, 43]. These may be useful in harm reduction service settings, e.g., supervised consumption spaces and syringe services, due to their ability to detect other drugs beyond fentanyl analogs, although these vibrational spectroscopy methods likely still require FTS augmentation to compensate for low sensitivity [57]. Mass spectrometry drug checking methods would likely be superior to IR and Raman spectroscopy methods and would not require FTS due to superior sensitivity [28]. The clear downsides of mass spectrometry are cost and access, although paper-spray mass spectrometry has been successfully implemented for fixed location drug checking in Canada [59]. FTS has the advantage in terms of cost, portability and adoptability, but their variation in performance and vulnerability to interferences may lead to false positives and false negatives without sample preparation instructions corresponding to the specific manufacturing lot.

Some limitations of these analyses should be noted. In this study, the 13 most cited fentanyl analogs were evaluated for cross-reactivity with the FTS; however, numerous additional fentanyl analogs exist, and their degree of cross-reactivity is still unknown. Additionally, only 11 illicit drugs and adulterants were evaluated for potential cross-reactivity and other untested substances could produce a false-positive FTS result. Determination of the FTS results by visual observation of the absence or presence of a line is subjective, which is a real-world limitation of their use and a potential limitation of the results of this study. All results were evaluated by 2 or more people in attempts to decrease subjectivity, but this process could be improved by recording individual reviewer analysis instead of only consensus decision to facilitate calculation of a Kappa statistic to summarize evaluator agreement. It is the nature of the FTS that not all results are clearly positive or negative.

## Conclusion

Drug checking has become an important aspect of harm reduction in the age of fentanyls. Expansion of these services is deemed essential albeit with concerns regarding capacity building, sustainability, and integration across services [60]. FTS is the most scalable drug checking technology, but challenges to implementation accuracy remain. The leading FTS, BTNX-20, demonstrated fentanyl sensitivity matching manufacturer claims for lots obtained in 2017, but more recent lots from 2021 were an order of magnitude less sensitive. Sensitivities for multiple fentanyl analogs also changed in either direction between the 2017 and 2021 lots. Relative to the 2017 lots, the 2021 lots had lower sensitivity for three analogs, most notably carfentanil, but improved sensitivity for four other analogs. There was no lot-to-lot variability for the two 2017 lots, but lot-to-lot variability was evident among the three 2021 lots, with sensitivities for analogs often different between the lots by one to two dilution steps.

The loss of fentanyl sensitivity from 2017 to 2021 was accompanied by an unwanted 10-to-100-fold increase in sensitivity for known interferences diphenhydramine, lidocaine, and MDMA. However, cross-reactivity for *d-*methamphetamine decreased to a threshold of 10 mg/mL, decreasing the chances of a false-positive result for fentanyl when testing methamphetamine. FTS is a scalable technology but limited in scope of information delivered. The analyses presented here support the continued use of FTS as part of an overdose prevention cascade that should include more sophisticated drug checking technologies. This study highlights the need for independent assessment of lot-to-lot performance of FTS and transparency regarding changes in reagents and manufacturing processes by FTS distributors.

### Supplementary Information

**Additional file 1.** Chemical structures of analytes. Chemical Structures of Fentanyl, select fentanyl analogs, and interferences evaluated in this study

**Additional file 2.** Fentanyl Test Strip Performance Detecting Fentanyl in Urine vs Water. Comparison of BTNX Rapid Response Fentanyl Test Strip (20 ng/mL cutoff) fentanyl sensitivity in urine compared to water. POS indicates a positive result, NEG indicates a negative result

**Additional file 3.** **Table 1A**. Example of estimated FTS results for a methamphetamine sample with no fentanyl contamination, dependent on drug check concentration. Drug check concentration refers to the concentration achieved when dissolving the drug sample (an unknown, impure mixture of multiple components) in water in preparation for FTS testing. **Table 1B**. Example of estimated FTS results for a methamphetamine sample with trace fentanyl contamination, dependent on drug check concentration. Drug check concentration refers to the concentration achieved when dissolving the drug sample (an unknown, impure mixture of multiple components) in water in preparation for FTS testing. **Table 1C**. Example of estimated FTS results for an MDMA sample with no fentanyl contamination, dependent on drug check concentration. Drug check concentration refers to the concentration achieved when dissolving the drug sample (an unknown, impure mixture of multiple components) in water in preparation for FTS testing. **Table 1D**. Example of estimated FTS results for an MDMA sample with trace fentanyl contamination, dependent on drug check concentration. Drug check concentration refers to the concentration achieved when dissolving the drug sample (an unknown, impure mixture of multiple components) in water in preparation for FTS testing.

## Data Availability

All data generated or analyzed during this study are included in this published article (and its supplementary information files).

## Abbreviations

FTS: Fentanyl test strip
IMF: Illicitly manufactured fentanyls

## References

[1] Ciccarone D (2019). The triple wave epidemic: supply and demand drivers of the US opioid overdose crisis. Int J Drug Policy.

[2] Spencer MR, Miniño AM, Warner M. Drug overdose deaths in the United States, 2001–2021. NCHS Data Brief No 457 Natl Cent Health Stat. 2022; 457. 36598401

[3] Diversion Control Division. National Forensic Laboratory Information System: NFLIS-Drug 2021 Annual Report. US Department of Justice, US Drug Enforcement Administration; 2022.

[4] Gladden RM, Martinez P, Seth P (2016). Fentanyl law enforcement submissions and increases in synthetic opioid-involved overdose deaths–27 states, 2013–2014. MMWR Morb Mortal Wkly Rep.

[5] O’Donnell JK, Halpin J, Mattson CL, Goldberger BA, Gladden RM (2017). Deaths involving fentanyl, fentanyl analogs, and U-47700–10 States, July–December 2016. MMWR Morb Mortal Wkly Rep.

[6] O’Donnell JK, Gladden RM, Seth P (2017). Trends in deaths involving heroin and synthetic opioids excluding methadone, and law enforcement drug product reports, by census region-United States, 2006–2015. MMWR Morb Mortal Wkly Rep.

[7] Unick GJ, Ciccarone D (2017). US regional and demographic differences in prescription opioid and heroin-related overdose hospitalizations. Int J Drug Policy.

[8] Shover CL, Falasinnu TO, Dwyer CL, Santos NB, Cunningham NJ, Freedman RB (2020). Steep increases in fentanyl-related mortality west of the Mississippi River: recent evidence from county and state surveillance. Drug Alcohol Depend.

[9] O’Donnell J. Notes from the field: opioid-involved overdose deaths with fentanyl or fentanyl analogs detected—28 states and the District of Columbia, July 2016–December 2018. MMWR Morb Mortal Wkly Rep. 2020; 69. https://www.cdc.gov/mmwr/volumes/69/wr/mm6910a4.htm 10.15585/mmwr.mm6910a4 PMC7075253 32163382

[10] Special testing and Research Laboratory. Drug Enforcement Administration Emerging Threat Report Mid-Year 2021. Drug Enforcement Administration; 2022.

[11] Mojica MA, Carter MD, Isenberg SL, Pirkle JL, Hamelin EI, Shaner RL (2019). Designing traceable opioid material§ kits to improve laboratory testing during the U.S. opioid overdose crisis. Toxicol Lett.

[12] Zhang Y, Halifax JC, Tangsombatvisit C, Yun C, Pang S, Hooshfar S, et al. Development and application of a High-Resolution mass spectrometry method for the detection of fentanyl analogs in urine and serum. J Mass Spectrom Adv Clin Lab. 2022; https://www.sciencedirect.com/science/article/pii/S2667145X22000232 10.1016/j.jmsacl.2022.07.005 PMC9440429 36065325

[13] Ciccarone D (2017). Fentanyl in the US heroin supply: a rapidly changing risk environment. Int J Drug Policy.

[14] Armenian P, Vo KT, Barr-Walker J, Lynch KL (2018). Fentanyl, fentanyl analogs and novel synthetic opioids: a comprehensive review. Neuropharmacology.

[15] Suzuki J, El-Haddad S (2017). A review: fentanyl and non-pharmaceutical fentanyls. Drug Alcohol Depend.

[16] Ciccarone D, Ondocsin J, Mars SG (2017). Heroin uncertainties: exploring users’ perceptions of fentanyl-adulterated and -substituted ‘heroin’. Int J Drug Policy.

[17] Carroll JJ, Marshall BDL, Rich JD, Green TC (2017). Exposure to fentanyl-contaminated heroin and overdose risk among illicit opioid users in Rhode Island: a mixed methods study. Int J Drug Policy.

[18] McKnight C, Des Jarlais DC (2018). Being “hooked up” during a sharp increase in the availability of illicitly manufactured fentanyl: adaptations of drug using practices among people who use drugs (PWUD) in New York City. Int J Drug Policy.

[19] Kral AH, Lambdin BH, Browne EN, Wenger LD, Bluthenthal RN, Zibbell JE (2021). Transition from injecting opioids to smoking fentanyl in San Francisco, California. Drug Alcohol Depend.

[20] Lambdin BH, Bluthenthal RN, Zibbell JE, Wenger L, Simpson K, Kral AH (2019). Associations between perceived illicit fentanyl use and infectious disease risks among people who inject drugs. Int J Drug Policy.

[21] Kandel DB, Hu MC, Griesler P, Wall M (2017). Increases from 2002 to 2015 in prescription opioid overdose deaths in combination with other substances. Drug Alcohol Depend.

[22] McCall Jones C, Baldwin GT, Compton WM (2017). Recent increases in cocaine-related overdose deaths and the role of opioids. Am J Public Health.

[23] Palamar JJ, Ciccarone D, Rutherford C, Keyes KM, Carr TH, Cottler LB (2022). Trends in seizures of powders and pills containing illicit fentanyl in the United States, 2018 through 2021. Drug Alcohol Depend.

[24] Twillman RK, Dawson E, LaRue L, Guevara MG, Whitley P, Huskey A (2020). Evaluation of trends of near-real-time urine drug test results for methamphetamine, cocaine, heroin, and fentanyl. JAMA Netw Open.

[25] Nolan ML, Shamasunder S, Colon-Berezin C, Kunins HV, Paone D (2019). Increased presence of fentanyl in cocaine-involved fatal overdoses: implications for prevention. J Urban Health.

[26] Brunt T. Drug Checking as a Harm Reduction Tool for Recreational Drug Users: Opportunities and Challenges. European Monitoring Centre for Drugs and Drug Addiction; 2017.

[27] Caudevilla F, Ventura M, Fornís I, Barratt MJ, Vidal C, lladanosa CG (2016). Results of an international drug testing service for cryptomarket users. Int J Drug Policy.

[28] Harper L, Powell J, Pijl EM. An overview of forensic drug testing methods and their suitability for harm reduction point-of-care services. Harm Reduct J. 2017 https://www.ncbi.nlm.nih.gov/pmc/articles/PMC5537996/10.1186/s12954-017-0179-5 PMC5537996 28760153

[29] Hondebrink L, Nugteren-van Lonkhuyzen JJ, Van Der Gouwe D, Brunt TM (2015). Monitoring new psychoactive substances (NPS) in The Netherlands: data from the drug market and the Poisons Information Centre. Drug Alcohol Depend.

[30] Fairbairn N, Coffin PO, Walley AY (2017). Naloxone for heroin, prescription opioid, and illicitly made fentanyl overdoses: challenges and innovations responding to a dynamic epidemic. Int J Drug Policy.

[31] Gilbert M, Dasgupta N (2017). Silicon to syringe: cryptomarkets and disruptive innovation in opioid supply chains. Int J Drug Policy.

[32] McGowan CR, Harris M, Platt L, Hope V, Rhodes T (2018). Fentanyl self-testing outside supervised injection settings to prevent opioid overdose: do we know enough to promote it?. Int J Drug Policy.

[33] Socías ME, Wood E (2017). Epidemic of deaths from fentanyl overdose. BMJ.

[34] Krieger MS, Yedinak JL, Buxton JA, Lysyshyn M, Bernstein E, Rich JD (2018). High willingness to use rapid fentanyl test strips among young adults who use drugs. Harm Reduct J.

[35] Sherman SG, Morales KB, Park JN, McKenzie M, Marshall BDL, Green TC (2019). Acceptability of implementing community-based drug checking services for people who use drugs in three United States cities: baltimore, Boston and Providence. Int J Drug Policy.

[36] Goldman JE, Waye KM, Periera KA, Krieger MS, Yedinak JL, Marshall BDL (2019). Perspectives on rapid fentanyl test strips as a harm reduction practice among young adults who use drugs: a qualitative study. Harm Reduct J.

[37] Peiper NC, Clarke SD, Vincent LB, Ciccarone D, Kral AH, Zibbell JE (2019). Fentanyl test strips as an opioid overdose prevention strategy: findings from a syringe services program in the Southeastern United States. Int J Drug Policy.

[38] Park JN, Frankel S, Morris M, Dieni O, Fahey-Morrison L, Luta M (2021). Evaluation of fentanyl test strip distribution in two Mid-Atlantic syringe services programs. Int J Drug Policy.

[39] Weicker NP, Owczarzak J, Urquhart G, Park JN, Rouhani S, Ling R (2020). Agency in the fentanyl era: exploring the utility of fentanyl test strips in an opaque drug market. Int J Drug Policy.

[40] Maghsoudi N, Tanguay J, Scarfone K, Rammohan I, Ziegler C, Werb D (2022). Drug checking services for people who use drugs: a systematic review. Addiction.

[41] Park JN, Sherman SG, Sigmund V, Breaud A, Martin K, Clarke WA (2022). Validation of a lateral flow chromatographic immunoassay for the detection of fentanyl in drug samples. Drug Alcohol Depend.

[42] BTNX INC. BTNX INC. Fentanyl Strips for Harm Reduction Use. 2022 [cited 2022 Jan 4]. BTNX INC. Fentanyl strips for harm reduction use. https://www.btnx.com/files/BTNX_Fentanyl_Strips_Harm_Reduction_Brochure.PDF

[43] Green TC, Park JN, Gilbert M, McKenzie M, Struth E, Lucas R (2020). An assessment of the limits of detection, sensitivity and specificity of three devices for public health-based drug checking of fentanyl in street-acquired samples. Int J Drug Policy.

[44] Bergh MSS, Øiestad ÅML, Baumann MH, Bogen IL (2021). Selectivity and sensitivity of urine fentanyl test strips to detect fentanyl analogues in illicit drugs. Int J Drug Policy.

[45] Lockwood TLE, Vervoordt A, Lieberman M (2021). High concentrations of illicit stimulants and cutting agents cause false positives on fentanyl test strips. Harm Reduct J.

[46] Wharton RE, Casbohm J, Hoffmaster R, Brewer BN, Finn MG, Johnson RC (2021). Detection of 30 fentanyl analogs by commercial immunoassay kits. J Anal Toxicol.

[47] DanceSafe. How to Test Your Drugs for Fentanyl. DanceSafe; 2020. https://dancesafe.org/wp-content/uploads/2020/10/DS-fentanly-instruction-2020.pdf

[48] Ciccarone D (2021). The rise of illicit fentanyls, stimulants and the fourth wave of the opioid overdose crisis. Curr Opin Psychiatry.

[49] Clark R. URGENT: Recent batch of fentanyl strips requires different dilutions|DanceSafe. 2021. https://dancesafe.org/urgent-most-recent-batch-of-fentanyl-test-strips-requires-more-dilution-when-testing-mdma-and-meth/

[50] Matsuda R, Rodriguez E, Suresh D, Hage DS (2015). Chromatographic immunoassays: strategies and recent developments in the analysis of drugs and biological agents. Bioanalysis.

[51] Baumann MH, Tocco G, Papsun DM, Mohr AL, Fogarty MF, Krotulski AJ (2020). U-47700 and its analogs: non-fentanyl synthetic opioids impacting the recreational drug market. Brain Sci.

[52] Delcher C, Wang Y, Vega RS, Halpin J, Gladden RM, O’Donnell JK (2020). Carfentanil Outbreak—Florida, 2016–2017. Morb Mortal Wkly Rep.

[53] Bhullar MK, Gilson TP, Singer ME (2022). Trends in opioid overdose fatalities in Cuyahoga County, Ohio: multi-drug mixtures, the African-American community and carfentanil. Drug Alcohol Depend Rep.

[54] Drug Enforcement Administration. 2020 Drug Enforcement Administration National Drug Threat Assessment. U.S. Department of Justice; 2021.

[55] Solomon N, Hayes J (2017). Levamisole: a high performance cutting agent. Acad Forensic Pathol.

[56] Dinwiddie AT. Notes from the Field: Antihistamine Positivity and Involvement in Drug Overdose Deaths—44 Jurisdictions, United States, 2019–2020. MMWR Morb Mortal Wkly Rep. 2022; 71. https://www.cdc.gov/mmwr/volumes/71/wr/mm7141a4.htm 10.15585/mmwr.mm7141a4 PMC9575478 36227774

[57] McCrae K, Tobias S, Grant C, Lysyshyn M, Laing R, Wood E (2020). Assessing the limit of detection of Fourier-transform infrared spectroscopy and immunoassay strips for fentanyl in a real-world setting. Drug Alcohol Rev.

[58] McCrae K, Tobias S, Stunden C. BCCSU drug checking operational technician manual version 2. British Columbia Centre on Sustance Use; 2022.

[59] Borden SA, Saatchi A, Vandergrift GW, Palaty J, Lysyshyn M, Gill CG (2022). A new quantitative drug checking technology for harm reduction: pilot study in Vancouver, Canada using paper spray mass spectrometry. Drug Alcohol Rev.

[60] Ciccarone D, Moran L, Outram S, Werb D (2023). Insights from drug checking programs: practicing bootstrap public health whilst tailoring to local drug user needs. Int J Environ Res Public Health.

