# Supplementary figures and images for: Testing the test strips: laboratory performance of fentanyl test strips
Source: Harm Reduct J. 2024 Jan 18;21:14. doi: 10.1186/s12954-023-00921-8 (PMC10795297; doi:10.1186/s12954-023-00921-8)

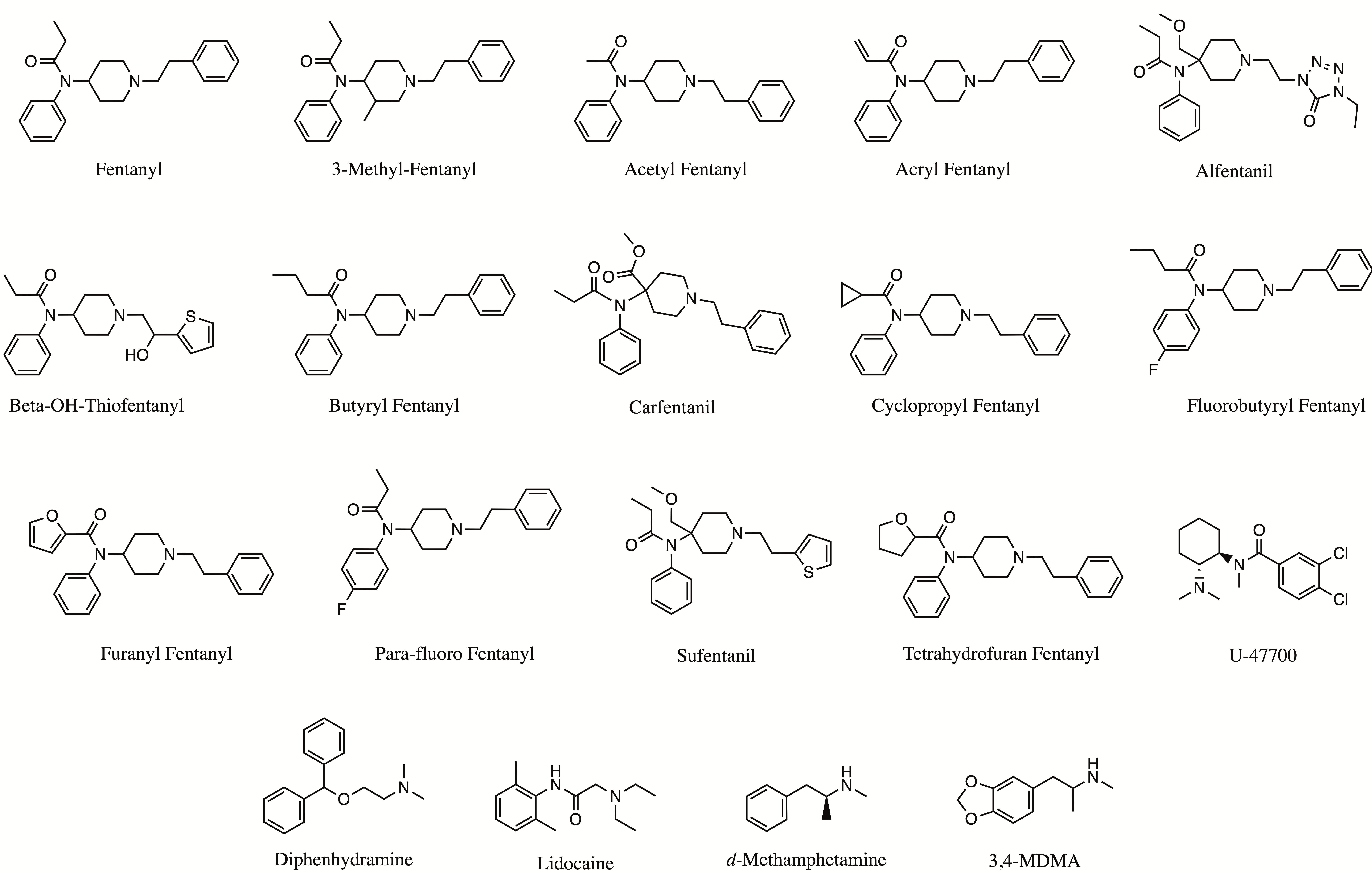

Supplement: Supplementary file 1 — Additional file 1. Chemical structures of analytes. Chemical Structures of Fentanyl, select fentanyl analogs, and interferences evaluated in this study [file 12954_2023_921_MOESM1_ESM.tiff]

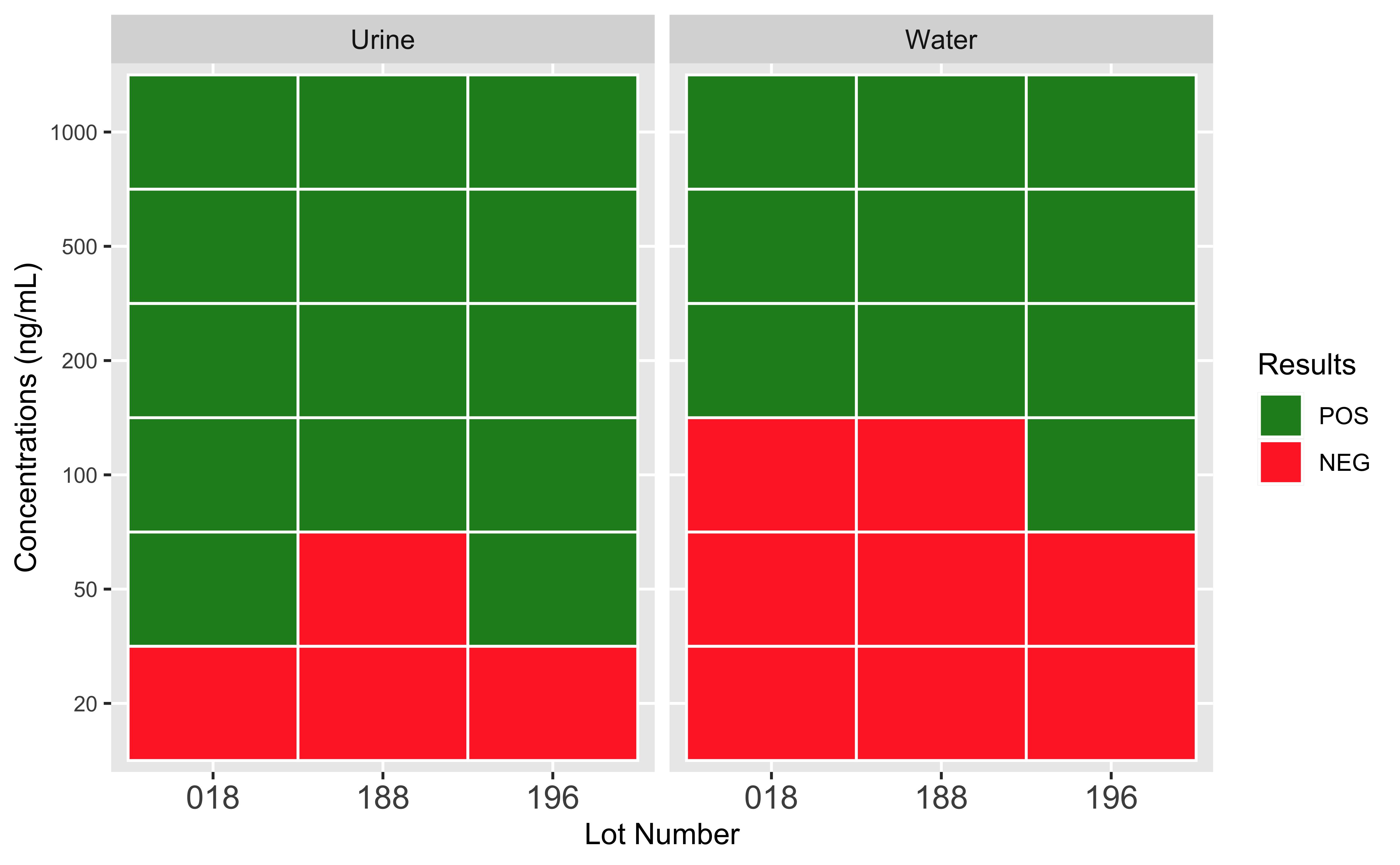

Supplement: Supplementary file 2 — Additional file 2. Fentanyl Test Strip Performance Detecting Fentanyl in Urine vs Water. Comparison of BTNX Rapid Response Fentanyl Test Strip (20 ng/mL cutoff) fentanyl sensitivity in urine compared to water. POS indicates a positive result, NEG indicates a negative result [file 12954_2023_921_MOESM2_ESM.tiff]
