# Supplementary material for: Testing the test strips: laboratory performance of fentanyl test strips
Source: Harm Reduct J. 2024 Jan 18;21:14. doi: 10.1186/s12954-023-00921-8 (PMC10795297; doi:10.1186/s12954-023-00921-8)
Supplement: Supplementary file 3 — Additional file 3. Table 1A. Example of estimated FTS results for a methamphetamine sample with no fentanyl contamination, dependent on drug check concentration. Drug check concentration refers to the concentration achieved when dissolving the drug sample (an unknown, impure mixture of multiple components) in water in preparation for FTS testing. Table 1B. Example of estimated FTS results for a methamphetamine sample with trace fentanyl contamination, dependent on drug check concentration. Drug check concentration refers to the concentration achieved when dissolving the drug sample (an unknown, impure mixture of multiple components) in water in preparation for FTS testing. Table 1C. Example of estimated FTS results for an MDMA sample with no fentanyl contamination, dependent on drug check concentration. Drug check concentration refers to the concentration achieved when dissolving the drug sample (an unknown, impure mixture of multiple components) in water in preparation for FTS testing. Table 1D. Example of estimated FTS results for an MDMA sample with trace fentanyl contamination, dependent on drug check concentration. Drug check concentration refers to the concentration achieved when dissolving the drug sample (an unknown, impure mixture of multiple components) in water in preparation for FTS testing. [file 12954_2023_921_MOESM3_ESM.docx]

| **Drug Check Concentration (mg/mL)** | **Sample Components**  **(% Purity by mass)** | **Component-Specific Concentrations** | **Estimated Result on 2017 Lots** | **Estimated Result on 2021 Lots** |
| --- | --- | --- | --- | --- |
| 15 mg/mL | Methamphetamine  (95%) | 14.25 mg/mL | **False Positive**  caused by methamphetamine | **False Positive**  caused by methamphetamine |
|  | Dimethyl Sulfone  (5%) | 750 µg/mL |  |  |
| 7 mg/mL | Methamphetamine  (95%) | 6.65 mg/mL | **False Positive**  caused by methamphetamine | **True Negative** |
|  | Dimethyl Sulfone  (5%) | 350 µg/mL |  |  |
| 2 mg/mL | Methamphetamine  (95%) | 1.9 mg/mL | **True Negative** | **True Negative** |
|  | Dimethyl Sulfone  (5%) | 100 µg/mL |  |  |

Supplementary Table 1A. Example of estimated FTS results for a methamphetamine sample with no fentanyl contamination, dependent on drug check concentration. Drug check concentration refers to the concentration achieved when dissolving the drug sample (an unknown, impure mixture of multiple components) in water in preparation for FTS testing.

| **Drug Check Concentration (mg/mL)** | **Sample Components**  **(% Purity by mass)** | **Component-Specific Concentrations** | **Estimated Result on 2017 Lots** | **Estimated Result on 2021 Lots** |
| --- | --- | --- | --- | --- |
| 15 mg/mL | Methamphetamine  (95%) | 14.25 mg/mL | **True Positive**  caused by fentanyl (also False Positive by methamphetamine) | **True Positive**  caused by fentanyl (also False Positive by methamphetamine) |
|  | Dimethyl Sulfone  (4.99%) | 748.5 µg/mL |  |  |
|  | Fentanyl  (0.01%) | 1.5 µg/mL |  |  |
| 7 mg/mL | Methamphetamine  (95%) | 6.65 mg/mL | **True Positive**  caused by fentanyl (also False Positive by methamphetamine) | **True Positive**  caused by fentanyl |
|  | Dimethyl Sulfone  (4.99%) | 349.3 µg/mL |  |  |
|  | Fentanyl  (0.01%) | 700 ng/mL |  |  |
| 2 mg/mL | Methamphetamine  (95%) | 1.9 mg/mL | **True Positive**  caused by fentanyl | **True Positive**  caused by fentanyl |
|  | Dimethyl Sulfone  (4.99%) | 99.8 µg/mL |  |  |
|  | Fentanyl  (0.01%) | 200 ng/mL |  |  |
| 1 mg/mL | Methamphetamine  (95%) | 950 µg/mL | **True Positive**  caused by fentanyl | **False Negative** |
|  | Dimethyl Sulfone  (4.99%) | 49.9 µg/mL |  |  |
|  | Fentanyl  (0.01%) | 100 ng/mL |  |  |

Supplementary Table 1B. Example of estimated FTS results for a methamphetamine sample with trace fentanyl contamination, dependent on drug check concentration. Drug check concentration refers to the concentration achieved when dissolving the drug sample (an unknown, impure mixture of multiple components) in water in preparation for FTS testing.

| **Drug Check Concentration (mg/mL)** | **Sample Components**  **(% Purity by mass)** | **Component-Specific Concentrations** | **Estimated Result on 2017 Lots** | **Estimated Result on 2021 Lots** |
| --- | --- | --- | --- | --- |
| 10 mg/mL | MDMA  (50%) | 5 mg/mL | **True Negative** | **False Positive**  caused by MDMA |
|  | Microcrystalline Cellulose  (50%) | 5 mg/mL |  |  |
| 2 mg/mL | MDMA  (50%) | 1 mg/mL | **True Negative** | **True Negative** |
|  | Microcrystalline Cellulose  (50%) | 1 mg/mL |  |  |

Supplementary Table 1C. Example of estimated FTS results for an MDMA sample with no fentanyl contamination, dependent on drug check concentration. Drug check concentration refers to the concentration achieved when dissolving the drug sample (an unknown, impure mixture of multiple components) in water in preparation for FTS testing.

| **Drug Check Concentration (mg/mL)** | **Sample Components**  **(% Purity by mass)** | **Component-Specific Concentrations** | **Estimated Result on 2017 Lots** | **Estimated Result on 2021 Lots** |
| --- | --- | --- | --- | --- |
| 10 mg/mL | MDMA  (50%) | 5 mg/mL | **True Positive**  caused by fentanyl | **True Positive**  caused by fentanyl (also False Positive by MDMA) |
|  | Microcrystalline Cellulose  (49.99%) | 4.999 mg/mL |  |  |
|  | Fentanyl  (0.01%) | 1 µg/mL |  |  |
| 2 mg/mL | MDMA  (50%) | 1 mg/mL | **True Positive**  caused by fentanyl | **True Positive**  caused by fentanyl |
|  | Microcrystalline Cellulose  (49.99%) | 999.8 µg/mL |  |  |
|  | Fentanyl  (0.01%) | 200 ng/mL |  |  |
| 1 mg/mL | MDMA  (50%) | 500 µg/mL | **True Positive**  caused by fentanyl | **False Negative** |
|  | Microcrystalline Cellulose  (49.99%) | 499.9 µg/mL |  |  |
|  | Fentanyl  (0.01%) | 100 ng/mL |  |  |

Supplementary Table 1D. Example of estimated FTS results for an MDMA sample with trace fentanyl contamination, dependent on drug check concentration. Drug check concentration refers to the concentration achieved when dissolving the drug sample (an unknown, impure mixture of multiple components) in water in preparation for FTS testing.
